# A gene-model-free method for linkage analysis of a disease-related-trait based on analysis of proband/sibling pairs

**DOI:** 10.1186/1471-2156-6-S1-S47

**Published:** 2005-12-30

**Authors:** Heejong Sung, Stephen J Finch, Kenny Q Ye, Nancy R Mendell

**Affiliations:** 1 Applied Mathematics and Statistics Department, Stony Brook University, Stony Brook, New York, 11794, USA; 2 Albert Einstein College of Medicine, Bronx, New York, 10461, USA

## Abstract

In this paper we investigate the power of finding linkage to a disease locus through analysis of the disease-related traits. We propose two family-based gene-model-free linkage statistics. Both involve considering the distribution of the number of alleles identical by descent with the proband and comparing siblings with the disease-related trait to those without the disease-related-trait. The objective is to find linkages to disease-related traits that are pleiotropic for both the disease and the disease-related-traits. The power of these statistics is investigated for Kofendrerd Personality Disorder-related traits a (Joining/founding cults) and trait b (Fear/discomfort with strangers) of the simulated data. The answers were known prior to the execution of the reported analyses. We find that both tests have very high power when applied to the samples created by combining the data of the three cities for which we have nuclear family data.

## Background

Because complex diseases are by definition determined by many genes and many environmental factors, unfeasibly large samples of nuclear families and affected relative pairs are needed to have reasonable power to detect linkage. More recently, attention has shifted to the analysis of endophenotypes, or disease-related traits (DRT). These traits are distributed differently in affected individuals than in controls and also are distributed differently distribution in siblings of affected individuals. This approach has been promising for several diseases. Examples are eye tracking disorder [1,2], a schizophrenia-related trait, and language deficits [3], an autism-related trait. In general it is hypothesized that the DRT might have a simpler etiology than the disease. In particular the disease may be caused by several genes and environmental factors, while the DRT may be caused only by one or two of the disease genes and fewer environmental factors [4].

The simulated data set gave us the opportunity to study a situation in which we have two binary DRTs, namely DRTa (Joining/founding cults) and DRTb (Fear/discomfort with strangers), which are determined by no more than two of the many genes that determine the disease phenotype, Kofendrerd Personality Disorder (KPD). In this research is a sample of families ascertained as a result of having at least one affected individual. The aim of this paper is to evaluate the power of statistics that compare DRT positive (DRT +) to DRT negative (DRT -) siblings of disease affected probands with respect to the number of alleles identical-by-descent to the proband (IBDP).

We conjecture that the disease and the DRT share some factor that is common to family members. When this factor is a gene, we expect that there would be differences in IBD when comparing sharing between DRT+ and DRT- siblings of the proband at the markers linked to the disease/DRT gene. Specifically we would expect D+/DRT+ sib pairs to be more alike in genotype at the disease/DRT locus and markers closely linked to disease/DRT than a D+/DRT- sib pairs.

In this paper we report the result of our analysis of two disease related traits using two statistical methods.

## Methods

### The data

We considered all 100 replicates. The data taken from each simulation consisted of all sib pairs in which at least one individual was affected. Thus with 300 families provided by combining the data from all three cities, we had information on about 750 proband/sib pairs. These data sets were generated as follows: 1) GENEHUNTER was run on all of the families and use the procedure "DUMP IBD" to obtain the IBD values for every relative pair in the sample. 2) All relative pairs that are not sib pairs were eliminated. That is, we kept only the data on those relative pairs in which the prior IBD values equal the values unique to sib pairs (0.25, 0.5, 0.25). 3) All sib pairs in which there are no individuals affected with the disease were eliminated.

In each sibship, there is at least one affected individual. This individual is designated as the *P *(proband). In the case where a family had two individuals affected by the disease, one is randomly assigned the designation of *P *and the other is considered as a *SP *(sib of the proband).

### DRTs and loci considered

We focused on DRTa (Joining/founding cults) and DRTb (Fear/discomfort with strangers) because they both resulted from no more than two of the many KPD genes. We considered all of the chromosome 1 loci because the answers indicated that there is one locus (D1) on this chromosome that is a dominant gene for both DRTa and DRTb. We used the typing for all markers on chromosome 1 given in the microsatellite data set.

### The variables analyzed

Each SP in the sample had data on the following variables for each genetic locus and DRT.

*Y *= the estimated IBDP = Z1 + 2.*Z*2   (1)

Here *Z*1 (*Z*2) are the values obtained from the GENEHUNTER analysis and denote the estimated posterior probability that *SP *and *P *share one(*Z*1) or two(*Z*2) alleles at the locus. We refer henceforth to *Y *as IBDP, the number of allele IBD to the proband. The second variable recorded was *DRTj*(*j *= *a,b*), where

*DRTj *= *DRT *+ if *SP *has the disease related trait *j*

         = *DRT *- if *SP *does not have the disease related trait *j *(2)

### Statistical tests

The *DRT *+ *SP *were compared to the *DRT *- *SP *using two test statistics: TLOD: The average value of *Y *in *DRT *+ *SP *() was compared to the average value of *Y *in *DRT *- *SP *() using a one-sided two sample with equal variance *t-test*. We then transformed the value of *T *to a value comparable to a LOD score value as follows:



Since *T *is distributed as a standard normal variable we need *TLOD > 3 *to have a value which is significant at the 0.0001 level one sided to the right. (Critical value of *T *for *α *= 0.0001 one sided is +3.71; 3.71^2 ^× 0.2171 = 3.0).

CLOD: Comparison of the distribution IBPD in the *DRT *+ *SP *to that in the *DRT *- *SP*. In this case the value of *Y *was rounded off to C*(Y) *as follows:

.

We then compared the distribution of C(*Y*) of the *DRT*+ to the *DRT*- using a Pearson chi-square test (*χ*^2^) for homogeneity of proportions for a 3 × 2 table. This statistic was also converted to a value comparable to the LOD score by computing

CLOD = *χ*^2^/(2 log_*e*_10) = 0.2171 × *χ*^2^   (4)

Since *χ*^2 ^is distributed as chi-square with 2 degrees of freedom (), we need a CLOD > 4 to have a value which is significant at the 0.0001 level. (Critical value for *α *= 0.0001 based on  distribution is 18.42068 ; 18.42 × 0.2171 = 4.0)

## Results

**Figure 1:**
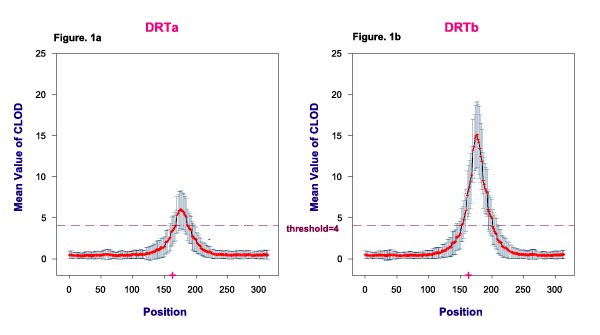
Mean value of CLOD for DRTa (a) and DRTb (b).

**Figure 2:**
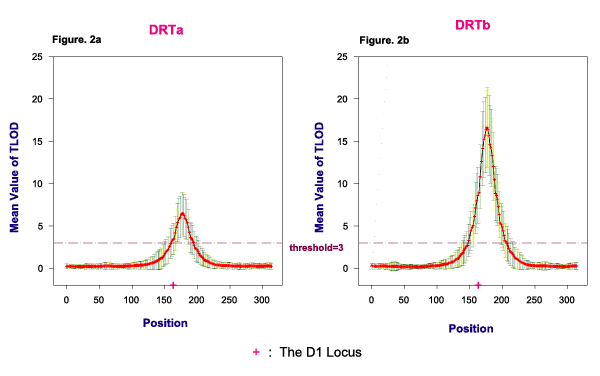
Mean value of TLOD for DRTa (a) and DRTb (b).

Figures 1 and 2 are the average values of CLOD (Figure 1) and TLOD (Figure 2) vs. position for *DRTa *(Figures 1a and 2a) and *DRTb *(Figures 1b and 2b). The shaded region around the plot represents the standard error of the mean. From the magnitude of the SE, we can see that we do not have precise estimates of the mean LOD. Specifically, the apparent peak average at position 177 is not significantly different from the values obtained at the markers on the interval from marker D01S0023 (160.428) to marker D01S0024 (167.428), the markers closest to the *D1 *locus, which is at position 163.

The average observed values of TLOD are well above 3.0 for all markers within 30 map units of the locus for DRTa and for all markers within 35 map units of the locus for DRTb. Similarly, the mean value of CLOD is well above 4.0 for all markers within 25 units of DRTa and all markers within 40 units of DRTb.

When we look at the each city individually, as expected, the mean LODs are not as high. The mean values of the test statistics in the regions of the D1 marker for DRT b vary considerably from city to city. Aipotu (highest mean CLOD = 4.06 and highest mean TLOD = 1.3) and Karangar (highest mean CLOD = 2.4 and highest mean TLOD = 0.7) seem to have a lower values than Danacaa (highest mean CLOD = 10.9 and highest mean TLOD = 3.2).

## Discussion

Both model-free methods have high estimated mean LODs at the DRT locus for *D1*. Upon considering these results in terms of power, we observe power of 100% for both tests in the analyses of DRTb and of 80% power in the analyses of DRTa. However, this is in part due to the enormous number of proband/sib pairs (about 750 pairs) available upon combining the data from the three cities. A second limitation of our results is that we used many more than one proband/sib pair per family. All sib of probands in our sample were used without taking into account the dependence of results obtained from sibs in the same family.

We conjecture that, depending on the genetic parameters, considering DRT alone may be as good as our method in some cases. The situations in which this approach is best need to be identified. However, this approach is quite straightforward and appears effective here. In a study considering both disease and DRT simultaneously using model-based genetic analysis [5], there were many situations when this approach was more powerful than considering just the DRT status.

The difference in power observed in the three cities cannot be explained by the differences in the sample size. It may be accounted for by the heterogeneity in the method of ascertaining cases and hence families of cases. The DRTb studied was determined by a locus that also was involved in determining KPD P1 (phenotype 1) and KPD P3 (phenotype 3). It was not involved in determining KPD P2 (phenotype 2). However, the family members of Aipotu were coded as KPD affected if they had P1, P2, or P3. Similarly, the family members of Karangar were coded as KPD affected if they had either P2 or P3. However, only in Danacaa were individuals required to have P1 to be designated as KPD affected. Thus, some of the KPD affecteds in Aipotu and Karanger did not have DRTb whereas all of the KPD affected individuals in Danacca had DRTb. Additionally, in Danaccaa we had less genetic heterogeneity than in the other two cities, and hence we had greater power.

The CLOD statistic is a family based Pearson chi-square test of homogeneity of distribution of IBD for a case-control study where the cases are *SP *who are *DRT *+ and the controls are *SP *who are *DRTa *-. Since the alternative distribution of TLOD is asymptotically normal and the alternative distribution of CLOD is asymptotically non-central chi-square, the power of both of these tests are functions of the genetic parameters for the disease/DRT locus and the number of proband/sib pairs observed. Knowledge of these functions could be extremely valuable in planning future studies. One would expect that the relative power of the two tests depend on the genetic generating model. It is not clear whether there are consistent differences in power across genetic models. If so, we may be able to recommend one of the two statistics at some future time. Here, we used both statistics since we had insufficient information on the underlying genetic model or the relative power of these tests. We would recommend at this point that investigators use both methods.

## Conclusion

1) We observed greater power to detect locus *D1*, through analysis of DRTb than DRTa. 2) Comparison of the distribution of alleles IBDP in DRTb+ siblings to DRTb- siblings resulted in excellent power (≥ 0.90) to detect locus *D1 *with 300 families. 3) The *t *test (TLOD) which compares the mean IBDP (number of alleles IBDP) of DRTb+ siblings to DRTb- siblings appears to be as powerful as the Pearson chi-square test (CLOD) comparing the distribution of IBDP of DRTb+ to DRTb-.

## Abbreviations

DRT: Disease-related-trait

CLOD: Pearson chi-squared statistic transformed to LOD scale

TLOD: *Two sample equal variance T test statistic transformed to LOD scale*

IBD: Identical by descent

IBDP: Number of alleles identical by descent to the proband

KPD: Kofendrerd Personality Disorder

## Authors' contributions

NRM, SJF, and KQY conceived of the study, and participated in its design and coordination and helped to draft the manuscript. NRM presented this work. HS carried out all of the analyses including the genetic analyses, data reduction, statistical analyses. SJF research is in part supported by NIMH grant number 2R01MH04480114A1.
